# A Case of Failure of EkoSonic™ Endovascular System in the Treatment of Submassive Pulmonary Embolism

**DOI:** 10.7759/cureus.15058

**Published:** 2021-05-16

**Authors:** Ekta Tirthani, Mina Said, Salem Thabet

**Affiliations:** 1 Internal Medicine, Rochester Regional Health, Rochester, USA

**Keywords:** ultrasound assisted catheter directed thrombolysis, ekos, submassive pulmonary embolism, half dose systemic thrombolytic therapy, failure of ekos

## Abstract

Submassive pulmonary embolism (SPE) is characterized by the presence of right ventricular (RV) strain as visualized on echocardiogram or CT scan with brain natriuretic peptide (BNP) and/or troponin elevation. The condition accounts for 20-25% of all pulmonary embolism (PE) cases. In cases of SPE, catheter-directed thrombolysis (CDT) is generally considered in the presence of severe hypoxemia, worsening RV dysfunction, patients with increasing tachycardia and elevated troponins, free-floating thrombus in the right atrium or RV, and presence of extensive clot burden. EkoSonic™ Endovascular System (EKOS; Boston Scientific, Marlborough, MA) has been successfully used to treat cases of PE even where systemic thrombolytic therapy has failed. However, in this article, we describe a unique case of the failure of EKOS in treating a 71-year-old African American man who presented to the hospital with progressively worsening chest pain, shortness of breath, and fatigue. He was suspected to have SPE; however, a CT pulmonary angiogram could not be performed to estimate the clot burden due to an acute kidney injury. He was diagnosed with coronavirus disease 2019 (COVID-19) pneumonia during the hospitalization and had a delayed EKOS procedure with minimal improvement in oxygenation and clot burden. He subsequently underwent half-dose systemic thrombolytic therapy with complete resolution of his symptoms. Given our success with half-dose systemic therapy, we propose that it may be considered as a “rescue therapy” in cases where EKOS fails to deliver results.

## Introduction

EkoSonic™ Endovascular System (EKOS; Boston Scientific, Marlborough, MA) catheter-directed thrombolysis (CDT) has been shown to be effective in reducing pulmonary arterial (PA) pressures and right ventricular (RV) strain in patients with submassive pulmonary embolism (SPE) faster than anticoagulation (AC) alone, thereby decreasing chances of hemodynamic decompensation as established by the following trials: Ultrasound Accelerated Thrombolysis of Pulmonary Embolism (ULTIMA); SEATTLE II; Pulmonary Embolism Response to Fragmentation, Embolectomy, and Catheter Thrombolysis (PERFECT); and OPtimum Duration and Dose of r-tPA with the Acoustic Pulse ThromboLYSis ProcEdure for Intermediate-Risk (Submassive) Pulmonary Embolism (OPTALYSE-PE) [1,2]. EKOS has been successfully used in cases of pulmonary embolism (PE) even when systemic thrombolytic therapy has failed [1,3,4]. We describe a unique case of failure of EKOS in a patient who was subsequently treated with half-dose systemic thrombolytic therapy (tPA) with complete resolution of symptoms.

## Case presentation

A 71-year-old African American man presented to the hospital with progressively worsening chest pain, shortness of breath, fatigue, and lightheadedness for one day. The chest pain was right-sided, non-radiating, and continuous with no exacerbating or relieving factors. He had a medical history of uncontrolled type 2 diabetes mellitus, morbid obesity, hypertension, obstructive sleep apnea (OSA), and tobacco abuse. He had undergone multiple joint replacement surgeries (the last one being three years before this presentation) and used crutches to ambulate. He was found to have blood pressure (BP) of 90/60 mmHg, heart rate (HR) of 110 beats per minute, respiratory rate (RR) of 35 breaths per minute, and SpO_2_ of 98% on room air. Physical exam revealed diaphoresis, respiratory distress, and decreased breath sounds in all lung fields. Labs revealed troponin of 0.91 ng/mL, brain natriuretic peptide (BNP) of 748 pg/ml, lactate of 5.0 mmol/L, creatinine of 1.5 mg/dl (baseline: 0.98), and D-dimer of >7,650 ng/mL; the severe acute respiratory syndrome coronavirus 2 (SARS-CoV-2) test was negative. Despite receiving fluid resuscitation, the patient remained persistently hypotensive and was transferred to the ICU due to a need for vasopressors.

**Figure 1:**
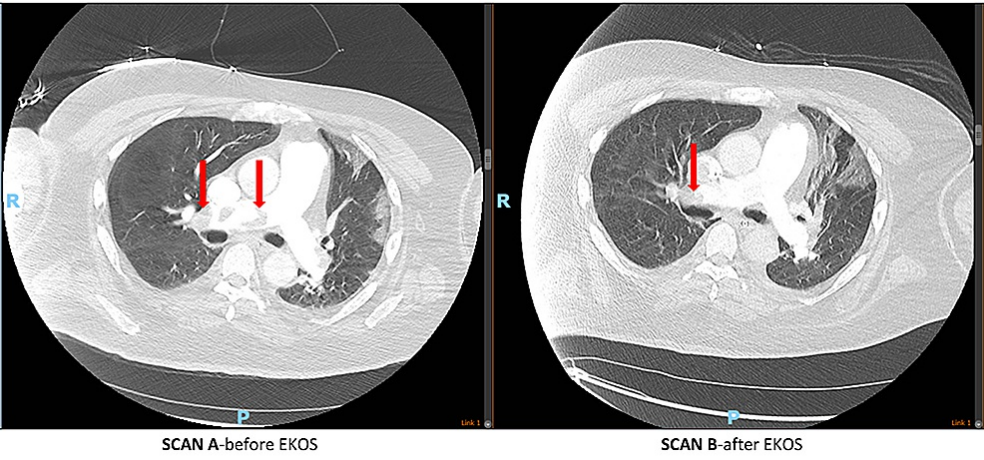
Minimal reduction in clot burden seen on CT chest pre and post EKOS procedure CT: computed tomography; EKOS: EkoSonic™ Endovascular System

A preliminary diagnosis of PE was made, but a CT pulmonary angiogram could not be performed due to an acute kidney injury. The patient had a bilateral lower extremity ultrasound, which showed acute right femoral deep vein thrombosis. An echo showed RV hypokinesis, flattened septum, and severe pulmonary hypertension. Thus, the diagnosis of SPE was confirmed, and systemic AC with a heparin drip was started. His blood pressure spontaneously improved within the same day, and he was transferred out of the ICU. However, over the next three days, his oxygen requirements went up again. SARS-CoV-2 test was repeated, which came back positive, raising the possibility of a false-negative test on admission versus hospital-acquired coronavirus disease 2019 (COVID-19) infection. At this point, he was transferred back to the ICU to administer high flow oxygen at 60 L and 100% FiO_2_, with a chest X-ray showing worsening bilateral infiltrates. His creatinine trended down from a peak of 2.6 mg/dl. A CT pulmonary angiogram was finally performed, which showed extensive bilateral PE with right and left lobar artery involvement and right heart strain. CDT with EKOS was carried out. However, the patient's FiO_2_ only minimally improved. A repeat CT pulmonary angiogram two days later showed a slight decrease in clot burden post EKOS with persistent right heart strain (Figure 1).

A repeat EKOS procedure was considered, but the clot appeared more organized on the new CT scan. The patient received half-dose systemic thrombolytic with tPA-50 mg over one hour with simultaneous AC. Within 24 hours, his oxygen requirements came down, and he was discharged home two days later.

## Discussion

The potential factors contributing to the failure of EKOS in our patient were as follows:

1. Delay in undergoing EKOS procedure (about six days after the diagnosis of SPE).

2. Inability to get a rapid estimation of clot burden with CT pulmonary angiogram due to an acute kidney injury.

3. COVID-19 infection-related hypercoagulability with an obstructive thrombo-inflammatory syndrome.

4. Given the patient's sedentary lifestyle, the presence of possible acute on chronic PE, which does not resolve with EKOS as thrombi get more organized over time.

The evidence for using systemic thrombolysis with half-dose tPA for SPE was established by the Moderate Pulmonary Embolism Treated With Thrombolysis (MOPETT) trial, which showed that half-dose tPA plus systemic AC decreased pulmonary hypertension incidence, PA pressures, and hospital stay [5]. Even the PERT Consortium guidelines suggest using low-dose tPA for SPE cases with evidence of clinical deterioration based on vital signs, the severity of RV dysfunction, tissue perfusion, and gas exchange in the absence of absolute contraindications to thrombolysis. Interestingly, in a retrospective analysis by Sharifi et al. about the use of half-dose tPA versus EKOS in 97 patients, half-dose tPA was found to decrease PA pressure more than EKOS, and this was found to be achieved in a shorter time and at a lower cost, primarily due to the reduction in the length of hospital stay [3]. So far, most studies have not discussed alternative therapies in scenarios where EKOS fails to successfully treat patients with SPE. Although more evidence is required, half-dose tPA might be considered a “rescue therapy” in cases where EKOS fails, as indicated by our success with this case.

## Conclusions

As catheter-directed therapy with EKOS continues to gain popularity due to its targeted action potential in treating SPE, one must be aware of alternative therapeutic options such as half-dose systemic thrombolytic therapy if patients fail to get better after undergoing the EKOS procedure.
